# Structural Basis for the Immunogenicity of the C-Terminus of VP1 of Echovirus 3 Revealed by the Binding of a Neutralizing Antibody

**DOI:** 10.3390/v14112322

**Published:** 2022-10-22

**Authors:** Shuai Qi, Wangjun Fu, Jinyan Fan, Li Zhang, Binyang Zheng, Kang Wang, Xiangxi Wang, Ling Zhu, Xinjian Li, Yuxia Zhang

**Affiliations:** 1 CAS Key Laboratory of Infection and Immunity, National Laboratory of Macromolecules, Institute of Biophysics, Chinese Academy of Sciences, Beijing 100101, China; 2 Department of Life Science, University of Chinese Academy of Sciences, Beijing 100049, China; 3 National Health Commission of the People’s Republic of China, Key Laboratory of Enteric Pathogenic Microbiology (Jiangsu Provincial Center for Disease Control and Prevention), Nanjing 210009, China; 4 Department of New Diseases, Changping Laboratory, Beijing 102206, China

**Keywords:** echovirus 3, monoclonal antibody (MAb), cryo-EM single particle analysis, structures, C-terminus of VP1, vaccine

## Abstract

Echovirus 3 (E3), a serotype of human enterovirus B (HEV-B), causes severe diseases in infants. Here, we determined the structures of E3 with a monoclonal antibody (MAb) 6D10 by cryo-EM to comprehensively understand the specificities and the immunological characteristic of this serotype. The solved cryo-EM structures of the F-, A-, and E-particles of E3 bound with 6D10 revealed the structural features of the virus–antibody interface. Importantly, the structures of E-particles bound with 6D10 revealed for the first time the nature of the C-terminus of VP1 for HEV-Bs at the structural level. The highly immunogenic nature of this region in the E-particles provides new strategies for vaccine development for HEV-Bs.

## 1. Introduction

Human enterovirus B (HEV-B), belonging to the human enterovirus species (HEV) in the *Picornaviridae* superfamily, is a non-enveloped virus with a positive single-stranded RNA (+ssRNA) genome. Members belonging to this family constitute more than 30 serotypes capable of causing diverse and acute human diseases such as carditis, encephalitis, meningitis, conjunctivitis, respiratory infections, poliomyelitis, and human hand, foot, and mouth disease [1,2]. In recent years, severe outbreaks of HEV-B infections have been documented in the USA, Europe, and Asia [3,4,5], but no vaccine or specific drugs are available against HEV-Bs or approved for antiviral therapy. Therefore, many studies aimed at delineating the structural basis for the pathogenesis of HEV-Bs have been undertaken to identify targets for drug design.

Our group has solved several structures of HEV-Bs, such as EV30 [6], EV-A71 [7,8], and Coxsackie virus B5 (CVB5) [9] using cryo-electron microscopy (EM) single-particle analysis. These structures unveil details of the nature of the crucial epitopes and their immunogenic features. Although the overall configurations of HEV-Bs are roughly similar for most members, there are some differences in their structures in the mesa, including the N- and C-terminus, BC loop and DE loop of VP1, the EF loop of VP2, and the EF loop and GH loop of VP3. The distinguishing segments, mostly located near the five-fold axes, are thought to contribute to the serotype-specific antigenic sites and form the “canyon”, the binding site for receptors, which allows HEV-Bs to enter inside host cells [6,7].

**Figure 1:**
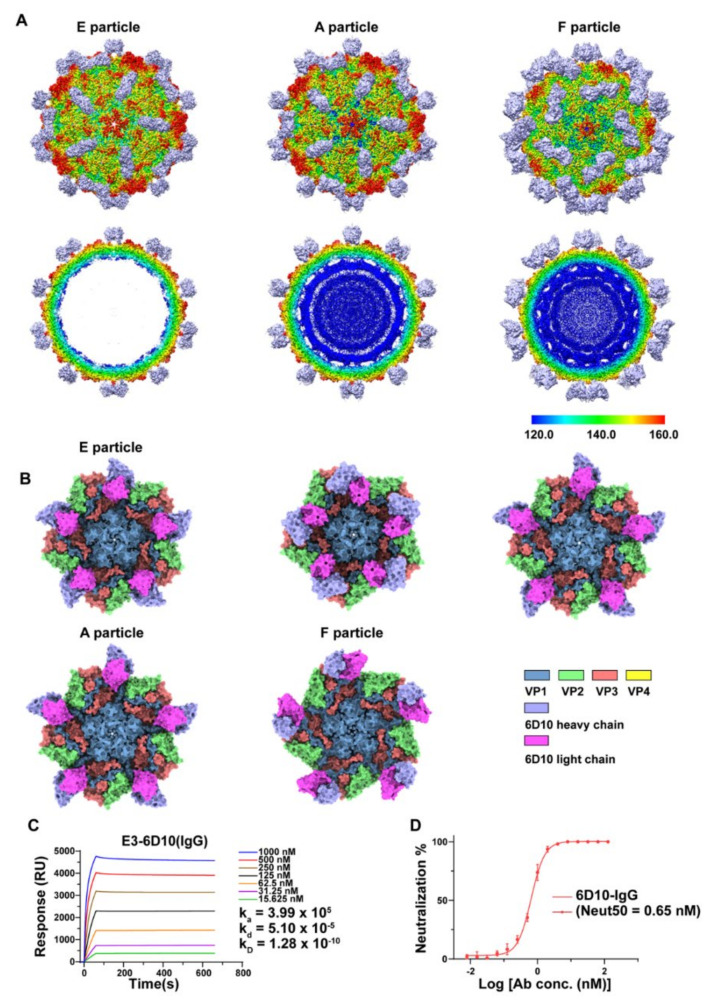
(**A**). Surface representation of the E3 F-, A-, and E-particles in complex with 6D10 and their corresponding central sections viewed along the twofold axes, respectively. The surface of the virus is colored by the radius from blue to red, and the Fab of the 6D10 is colored in light purple. (**B**). The top view of the 6D10 binding on a viral pentamer. The color scheme is shown at the bottom of the panel. (**C**). The binding affinity of 6D10 IgG with E3 was estimated by surface plasmon resonance (SPR). (**D**). Neutralization curve of the interaction of E3 with 6D10 measured by the plaque-reduction neutralization test (PRNT).

Similar to other HEV-Bs, the genome of E3 comprises a single open reading frame (ORF) made up of approximately 7500 nucleotides. The ORF codes for a polyprotein, which is processed post-translationally to generate the structural proteins (i.e., VP1-VP4) and the nonstructural proteins. In our previously published study, we described the overall structure of E3. In addition, we identified three types of particles of E3. The full particle, also referred to as F-particle, contained a significant amount of viral RNA. Therefore, it could be considered a representative of the mature virus. In contrast to the F-particle, no density for RNA was observed in the other two particles. These particles, devoid of RNA, were classified into two different types based on their size inferred from the maps. The particles were denoted as expanded (EE-) particles and compact empty (EC-) particles [10]. To comprehensively understand the basis for the specificities of the serotypes and further explore the immunological characteristics of E3 as an extension of our previous work, a monoclonal antibody (MAb) named 6D10 was generated by immunizing mice in this study. Here, we describe the structural analysis of the complex of E3-6D10 determined by cryo-EM (Figure 1A,B). The E3 virus (genotype HNWY-01 strain) used in this study was obtained from the Jiangsu Provincial Center for Disease Control and Prevention (CDC) and propagated in Rhabdomyosarcoma (RD) cells, as previously described [10].

## 2. Method

### 2.1. E3 Production and Purification

The production and purification of echovirus 3 (E3) were carried out as previously described [10]. In brief, E3, obtained from the Jiangsu Provincial Center of Disease Control and Prevention, China, as a gift, was used to infect Rhabdomyosarcoma (RD) cells at a multiplicity of infection (MOI) of 0.001 at 37 °C. The cells were cultured in Dulbecco’s modified Eagle’s medium (DMEM; Sigma) supplemented with 0.5% fetal bovine serum (FBS) (Gibco). The cells were harvested after 48 h infection, lysed with 3 freeze–thaw cycles to disrupt the cells in PBS buffer (pH 7.4) with 1% NP-40 solution, and centrifuged to remove the cell debris. The supernatant was passed through a 0.22 μm filter, concentrated with a 100 kD cutoff concentrator, and washed with PBS buffer repeatedly. Viral particles were pelleted by 20% (*w*/*v*) sucrose in PBS cushion centrifugation. The concentrated Echovirus 3 was loaded onto a 15–45% (*w*/*v*) sucrose density gradient and centrifuged at 29,000 rpm for 5 h. Two sets of fractions were collected and dialyzed against PBS buffer. 

### 2.2. Monoclonal Antibody 6D10 Preparation

The purified E3 virus F particles were used to immunize 10-week-old BALB/c female mice via hypodermic injection, with an amount of 50 μg/100 μL per mouse. The mice were boosted three times at 15 days, 30 days, and 45 days after the first injection. Five days after the final boost immunization (without CpG), the splenocytes of the mice were taken out and fused to SP2/0 myeloma cells using 50% polyethylene glycol (PEG) 4000 (Sigma-Aldrich, St Louis, MO, USA). The cells were seeded into 96-well plates containing Hypoxanthine/Aminopterin/Thymidine (HAT) (Sigma-Aldrich, St Louis, MO, USA) medium, which were seeded with feeder cells (peritoneal macrophages) 24 h before. The hybridomas’ culture supernatants were screened by ELISA, in which the E3 particles were coated as an antigen and the selected hybridomas were treated as a primary antibody. The positive clones were mixed with E3 virions (the plaque-forming unit (PFU) ranged from 50 to 100) at 37 °C for 1 h and then added onto the RD cell monolayers to screen the ones that could neutralize E3 by the plaque-reduction neutralization test (PRNT) as described below.

### 2.3. Plaque-Reduction Neutralization Assay

The plaque-reduction neutralization assay was carried out as previously described. In brief, the 6D10 IgG was diluted with DMEM to obtain twofold serial dilutions, generating 15 group samples in total with the highest concentration at 128 nM. The same amounts of E3 virions (PFU= 90) were mixed with the prepared dilutions or PBS as a control incubated at 37 °C for 1 h and dropped onto the confluent monolayers of RD cells seeded in 6-well plates. The plates were incubated in a 5% CO_2_ incubator with gentle shaking every 20 min for 1 h and then washed with DMEM (pH 7.4) three times. Thereafter, each well was covered by a 2 mL agarose overlay with 2% FBS and incubated in an incubator for 72 h. The plaques were visualized by staining with 2.5% crystal violet, and the percent inhibition was calculated as (Ncontrol−Ntest)/Ncontrol × 100%, where Ncontrol and Ntest represent the mean of plaque counts observed in the control group and test groups, respectively.

### 2.4. Surface Plasmon Resonance

The purified E3 F-particles were immobilized onto the surface of a CM5 sensor chip with PBS running buffer containing 0.05% Tween-20 and then applied to a Bia-core T100 (GE Healthcare, Piscataway, NJ, USA). The purified 6D10 IgG flew over the chip at a rate of 20 μL/min with gradient concentrations (i.e., 15.625, 31.25, 62.5, 125, 250, 500, and 1000 nM). The chip was regenerated by 10 mM glycine-HCl (pH 1.5) after each cycle. The response of 6D10 to the E3 virions was recorded at room temperature, and the data analysis was performed using Bia-core T100 Evaluation Software version 2.0 (GE Healthcare, Piscataway, NJ, USA).

### 2.5. 6D10 Fab Fragment Purification

The 6D10 Fab fragment was prepared using the Pierce FAB preparation Kit (Thermo Scientific, MA, USA) following the manufacturer’s instructions as previously described [10]. In brief, following removal of the salt with a desalting column, the antibody was mixed with papain and incubated for digestion at 37 °C for 3–4 h. The 6D10 Fab fragments were separated from the Fc fragments using a protein A affinity column and then concentrated for further experiments. The 6D10 sequences are provided in Supplementary Table S3. 

### 2.6. Grid Preparation

To prepare the E3-6D10 complex, purified E3 particles were treated with 6D10 Fab fragments at a ratio of 1:180 for 10 s incubations on ice. The complex of E3-6D10 samples (3 μL) was applied to a freshly glow-discharged 400 mesh Quantifoil 1.2/1.3 carbon-coated copper grid (C-flat, CF-2/1-2C, Protochips) using a Vitrobot Mark IV instrument (FEI) with a blotting time of 3 sec under 100% humidity at 4 °C, and the grids were plunge-frozen in liquid ethane. 

### 2.7. Cryo-EM Data Collection and Image Processing

The prepared grids were transferred to an Arctica electron microscope (FEI), which was operated at 200 kV and equipped with a Gatan Quantum-LS Energy Filter (GIF) and a Gatan K2 Summit direct electron detector in the electron counting mode with a defocus range of 1.2 to 2.5 um. Imaging was performed at a calibrated pixel size of 1.35 Å. Each movie was recorded for 0.2 s and subdivided into 25 frames with a total dose of 30 e-/A2.

The detailed information is described in Supplementary Table S2. For all datasets, dose-fractionated movies were subjected to beam-induced motion correction using Relion 3 [11], and the contrast transfer function (CTF) parameters were estimated using GCTF.

Particles were extracted from motion-corrected micrographs with downsampling to a pixel size of 2.7 Å/pixel. These particles were subjected to one round of 2D classification and two rounds of 3D classification (Figure S1). The best particles were selected, re-extracted with a pixel size of 1.35 Å/pixel, and subjected to auto-3D refinement, yielding F-, A-, and E-particles. Next, BLOCK-based reconstruction [12] was used to achieve clearer density for the interfaces of the complexes by symmetry expanding and re-extracting the protomers and MAb region and subsequently returned to the virus particles and performed refinement for the complexes of E3 particles with 6D10. Except for the E-particle, which was classified into two conformations, the other two contained only one uniform conformation at this stage. The global resolutions for the F-, A-, and E-particles were 2.8 Å, 3.2 Å, and 3.2 Å, respectively. All resolutions were calculated according to the gold-standard Fourier shell correlation (FSC) = 0.143 criterion. The detailed processing strategies are shown in Supplementary Figures S1–S3 and Supplementary Table S2.

### 2.8. Model Building, Refinement, and Validation

The cryo-EM structures of E3-6D10 were modeled using antibodies 305-78-7 (PDB ID: 5HDQ) and NC10 (PDB ID: 1NMC) as the initial models. After manual modification of the models by COOT [13], structure refinement was carried out using ‘phenix.real_space_refine’ in PHENIX with secondary structure restraint [11]. The geometry of the structure models was validated using MolProbity [14]. All the structural Figures were prepared in UCSF Chimera [15].

## 3. Results

**Figure 2:**
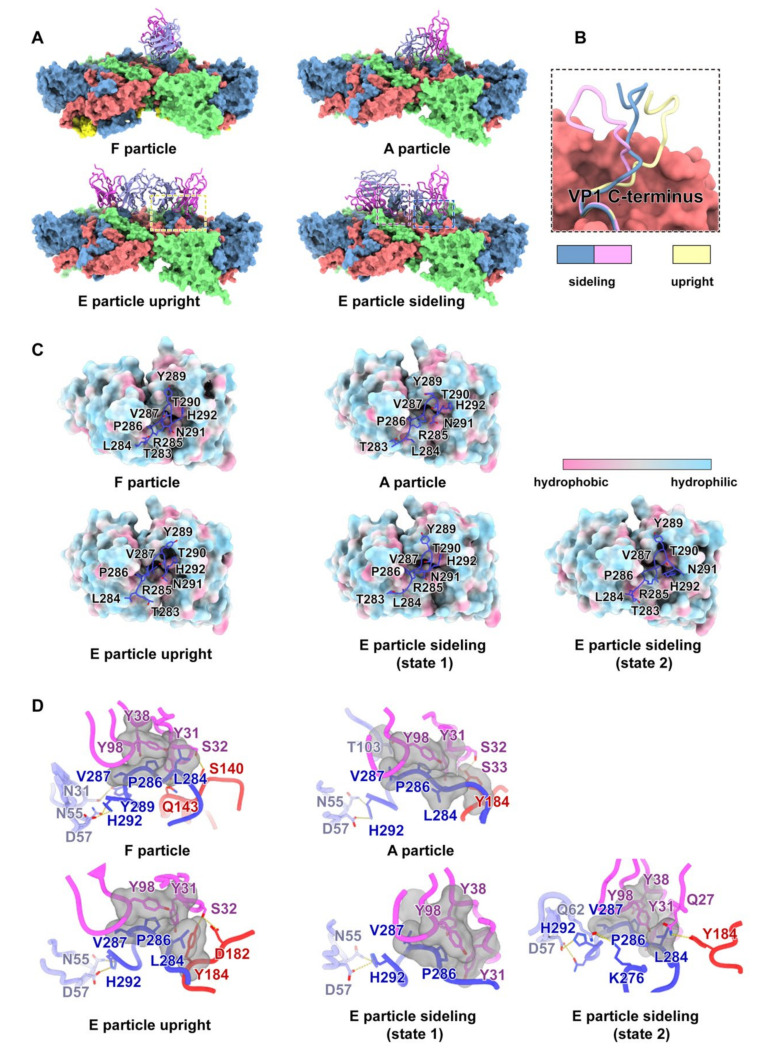
(**A**). Side view of the binding model of the E3–6D10 complex. The E3 is represented by the surface, and 6D10 is shown as a cartoon. The color scheme is the same as in panel B. The dotted squares indicate the region in panel F. (**B**). Superimposition of the C-terminus of VP1 in the E-upright and E-sideling particles. The C-terminus of VP1 is highlighted in cartoon, and the surface indicates VP3. (**C**,**D**). The upper panels (panel G) represent the VP1 C-terminal (shown as dark blue sticks) embedded into the hydrophobic pocket of the 6D10 (colorful surface). The lower panels (panel H) display the detailed interactions between the E3 particles and 6D10 corresponding to panel G. The key residues are shown as sticks. Hydrogen bonds are marked as yellow dashes. The color scheme is the same as in panel B.

The MAb 6D10 displayed high binding affinity to E3 with a K_D_ value of 0.13 nM (Figure 1C) as measured by surface plasmon resonance (SPR). Furthermore, it also showed a potent neutralizing activity against E3 with a Neut_50_ (50% neutralizing concentration) value of ~ 0.65 nM (Figure 1D). The results of our cryo-EM studies revealed 6D10 bound to three different types of E3 particles, the mature full (F-) particle, the intermediate altered (A-) particle, and the procapsid empty (E-) particle, resulting in three different maps with 2.8-, 3.2-, and 3.2-Å resolutions, respectively (Figure 1A,B) (Figures S1 and S2) (Table S2). With the help of the newly developed “block-based” algorithm (Figure S3A) [12], which is a powerful reconstruction method for resolution improvement, especially for the flexible regions of large protein complexes or viruses, we achieved clearer density at the interfaces between E3 and 6D10 in the maps (Figure S2A–C). According to the structure of the complex, the 6D10 attaches itself to the viral surface along the edges of the pentameric building blocks of the virus and adjacent to the twofold axes, a binding location similar to that of our recently reported antibody MAb 5G3 [10]. The variable domains of 6D10 recognized two epitopes of the E3 F-particle, including the C-terminus of VP1 (residues L284, P286, V287, Y289, and H292) and the EF loop of VP3 (residues S140 and Q143) at the interface (Figure 2C,D, upper left panel) (Table S1). Although the A-particle exhibited a significantly expanded structure, with an approximately 4.5% larger particle size than the F-particle, the 6D10 contacted the A-particle at very similar locations to those observed for the F-particle (Figure 2A, upper panels). The E-particle expanded the same size in capsid diameter as the A-particle (Figure 1A,B). Notably, two units of 6D10 bound the E-particle near the icosahedral twofold axes (Figure 2A, lower panels), which was different from other HEV-Bs, indicating that the E-particle was more immunogenic than the other two particles and could possibly serve as a promising candidate for the development of a vaccine. More interestingly, we obtained two distinctive conformations for the E-particle with an additional round of 3D classification, named E-upright and E-sideling. The differences in the position of the binding of 6D10 seem to have given rise to the two conformers (Figure S1 and S2A–C). Although the overall mode of the binding of the 6D10 to the E-upright and E-sideling particles at the C-terminus of VP1 and the EF loop of VP3 was comparable to those observed for the F- and A-particles (Figure 2C,D), the 6D10 shifted and moved away from the twofold axes while binding to the E-sideling, which may have been caused by the flexible nature of the C-terminus of VP1, resulting in the rearrangement of the structural elements (Figure 2B). Such a rearrangement of VP1 probably created even more steric hindrance and interfered with the binding of the virus with its receptors, because a comparison of the footprint of the 6D10 on E3 suggested that the locations of the paratopes of 6D10 were very close to but had no significant overlap with the binding sites of receptors such as FcRn and CD55 of HEV-Bs (Figure S3B). These structural insights seem to suggest that the expanded and loose configuration of the E-particle allows the C-terminus to be unburied and exposed from beneath, reducing steric hindrance and creating space for the binding of one more MAb near the twofold axes. Furthermore, these structural observations also suggest that the C-terminus of VP1 plays an essential role in the immunogenicity for E3. Coincidently, several studies have proposed that the C-terminus of VP1, the most diverse in sequence among the HEV-Bs, such as, Coxsackievirus A9 (CAV9) [16] and E22, plays a critical role in receptor recognition and is important for infectivity [17].

## 4. Conclusions

The cryo-EM structures of the F-, A-, and E-particles of E3 bound with 6D10 revealed the structural features of the virus–antibody interface. Importantly, our structures of E-particles bound with 6D10 revealed for the first time the nature of the C-terminus of VP1 for HEV-Bs at the structural level, which to date remains unidentified for most picornavirus due to its flexible nature and largely disordered properties. Our structural studies further underscore the importance of the VP1 C-terminus of the E-particle in the pathogenesis of the virus. In addition, the highly immunogenic nature of this region in the E-particles makes them good candidates for the development of a vaccine. The results presented here also provide a framework for the design of therapeutics targeting E3 and other echovirus family members.

## Data Availability

The atomic coordinates of E3 F-6D10, A-6D10, E-upright, and E-sideling complexes have been deposited in the Protein Data Bank under accession codes: 8GSD, 8GSC, 8GSE and 8GSF, respectively. Their corresponding cryo-EM density maps have been deposited in the Electron Microscopy Data Bank under accession codes: EMD-34232, EMD-34231, EMD-34233, and EMD-34234, respectively.

## Supplementary Materials

The following supporting information can be downloaded at: https://www.mdpi.com/article/10.3390/v14112322/s1, Figure S1: Workflow of data processing for cryo-EM single-particle analysis of Enterovirus 3 (E3) with its neutralizing antibody 6D10; Figure S2: A–C. Cross-validation FSC curves for map-to-model fitting and the interfaces of E3 F-, A-, and E-particles with 6D10, respectively. VP1, VP2, and VP3 from one protomer are colored blue, green and red, respectively; Figure S3: A. Data processing of E3-6D10 by BLOCK to improve the density at the interface; Table S1: Interaction of E3 with 6D10; Table S2: Cryo-EM data collection and refinement statistics; Table S3: Sequence of 6D10.

## References

[1] Chang L.-Y., Lin T.-Y., Hsu K.-H., Huang Y.-C., Lin K.-L., Hsueh C., Shih S.-R., Ning H.-C., Hwang M.-S., Wang H.-S. (1999). Clinical features and risk factors of pulmonary oedema after enterovirus-71-related hand, foot, and mouth disease. Lancet.

[2] Palacios G., Oberste M. (2005). Enteroviruses as agents of emerging infectious diseases. J. Neurovirol..

[3] Pogka V., Emmanouil M., Labropoulou S., Voulgari-Kokota A., Angelakis E., Mentis A.F. (2020). Molecular characterization of enteroviruses among hospitalized patients in Greece, 2013–2015. J. Clin. Virol..

[4] Huang Y.-P., Lin T.-L., Lin T.-H., Wu H.-S. (2017). Molecular and epidemiological study of enterovirus D68 in Taiwan. J. Microbiol. Immunol. Infect..

[5] He Y.-Q., Chen L., Xu W.-B., Yang H., Wang H.-Z., Zong W.-P., Xian H.-X., Chen H.-L., Yao X.-J., Hu Z.-L. (2013). Emergence, circulation, and spatiotemporal phylogenetic analysis of coxsackievirus a6-and coxsackievirus a10-associated hand, foot, and mouth disease infections from 2008 to 2012 in Shenzhen, China. J. Clin. Microbiol..

[6] Wang K., Zhu L., Sun Y., Li M., Zhao X., Cui L., Zhang L., Gao G.F., Zhai W., Zhu F. (2020). Structures of Echovirus 30 in complex with its receptors inform a rational prediction for enterovirus receptor usage. Nat. Commun..

[7] Wang X., Peng W., Ren J., Hu Z., Xu J., Lou Z., Li X., Yin W., Shen X., Porta C. (2012). A sensor-adaptor mechanism for enterovirus uncoating from structures of EV71. Nat. Struct. Mol. Biol..

[8] Zhu L., Xu K., Wang N., Cao L., Wu J., Gao Q., Fry E.E., Stuart D.I., Rao Z., Wang J. (2018). Neutralization mechanisms of two highly potent antibodies against human enterovirus 71. MBio.

[9] Yang P., Shi D., Fu J., Zhang L., Chen R., Zheng B., Wang X., Xu S., Zhu L., Wang K. (2022). Atomic Structures of Coxsackievirus B5 Provide Key Information on Viral Evolution and Survival. J. Virol..

[10] Feng R., Wang L., Shi D., Zheng B., Zhang L., Hou H., Xia D., Cui L., Wang X., Xu S. (2021). Structural basis for neutralization of an anicteric hepatitis associated echovirus by a potent neutralizing antibody. Cell Discov..

[11] Afonine P.V., Poon B.K., Read R.J., Sobolev O.V., Terwilliger T.C., Urzhumtsev A., Adams P.D. (2018). Real-space refinement in PHENIX for cryo-EM and crystallography. Acta Crystallogr. Sect. D Struct. Biol..

[12] Zhu D., Wang X., Fang Q., Van Etten J.L., Rossmann M.G., Rao Z., Zhang X. (2018). Pushing the resolution limit by correcting the Ewald sphere effect in single-particle Cryo-EM reconstructions. Nat. Commun..

[13] Emsley P., Cowtan K. (2004). Coot: Model-building tools for molecular graphics. Acta Crystallogr. Sect. D Biol. Crystallogr..

[14] Chen V.B., Arendall W.B., Headd J.J., Keedy D.A., Immormino R.M., Kapral G.J., Murray L.W., Richardson J.S., Richardson D.C. (2010). MolProbity: All-atom structure validation for macromolecular crystallography. Acta Crystallogr. Sect. D Biol. Crystallogr..

[15] Pettersen E.F., Goddard T.D., Huang C.C., Couch G.S., Greenblatt D.M., Meng E.C., Ferrin T.E. (2004). UCSF Chimera—A visualization system for exploratory research and analysis. J. Comput. Chem..

[16] Leippert M., Pfaff E. (2001). Foot-and-mouth disease virus can utilize the C-terminal extension of coxsackievirus A9 VP1 for cell infection. J. Gen. Virol..

[17] Stanway G., Kalkkinen N., Roivainen M., Ghazi F., Khan M., Smyth M., Meurman O., Hyypiä T. (1994). Molecular and biological characteristics of echovirus 22, a representative of a new picornavirus group. J. Virol..

