# Supplementary material for: Structural Basis for the Immunogenicity of the C-Terminus of VP1 of Echovirus 3 Revealed by the Binding of a Neutralizing Antibody
Source: Viruses. 2022 Oct 22;14(11):2322. doi: 10.3390/v14112322 (PMC9692665; doi:10.3390/v14112322)
Supplement: Supplementary file 1 [file viruses-14-02322-s001.zip › viruses-1939094-supplementary.pdf]

viruses

MDPI

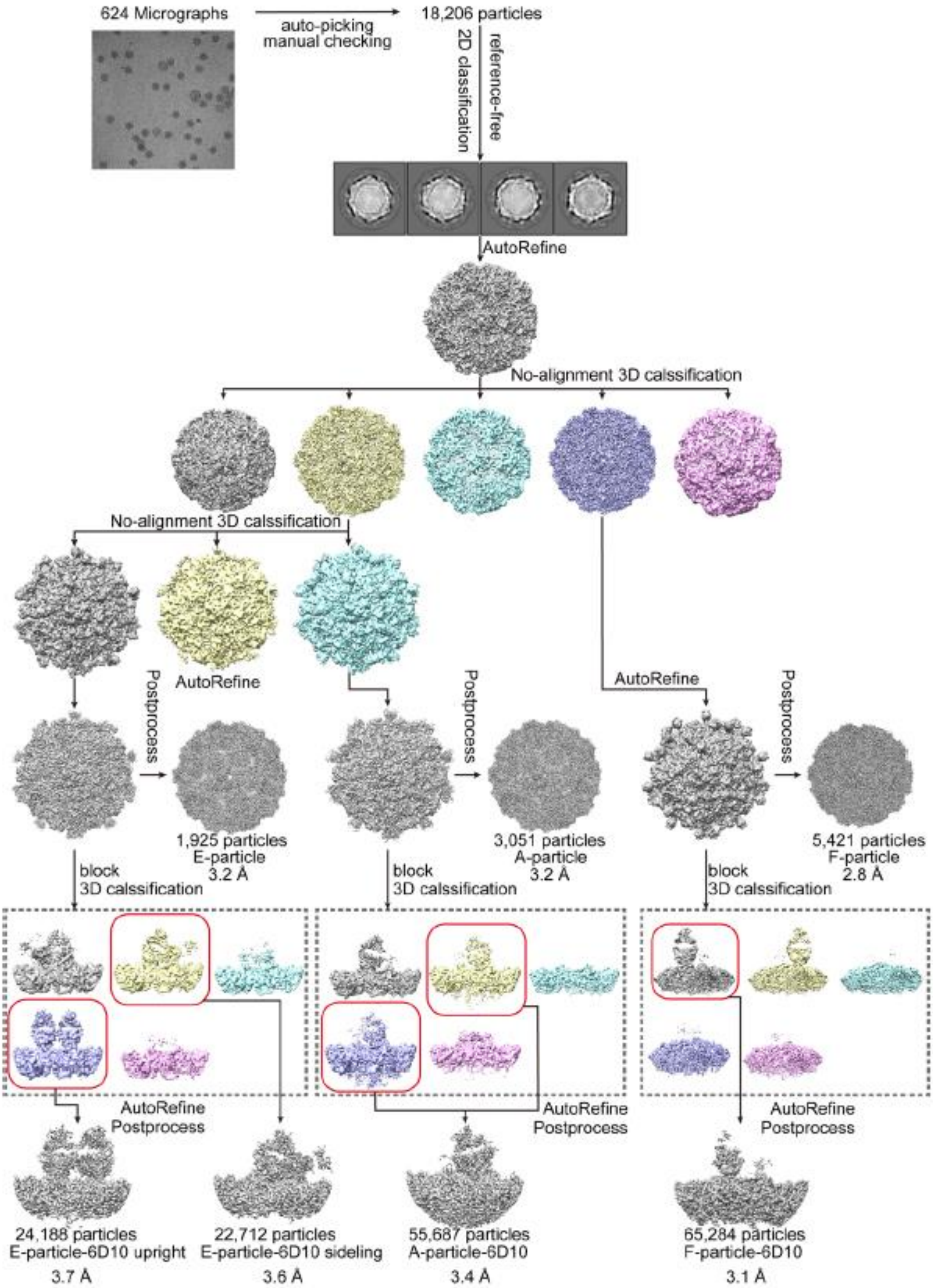

Figure S1. Workflow of data processing for cryo-EM single-particle analysis of Enterovirus 3 (E3) with its neutralizing antibody 6D10. The gray dotted squares indicate the models in one group generated by BLOCK analysis. The best maps selected for final auto-refine and postprocess were shown in the red squares.

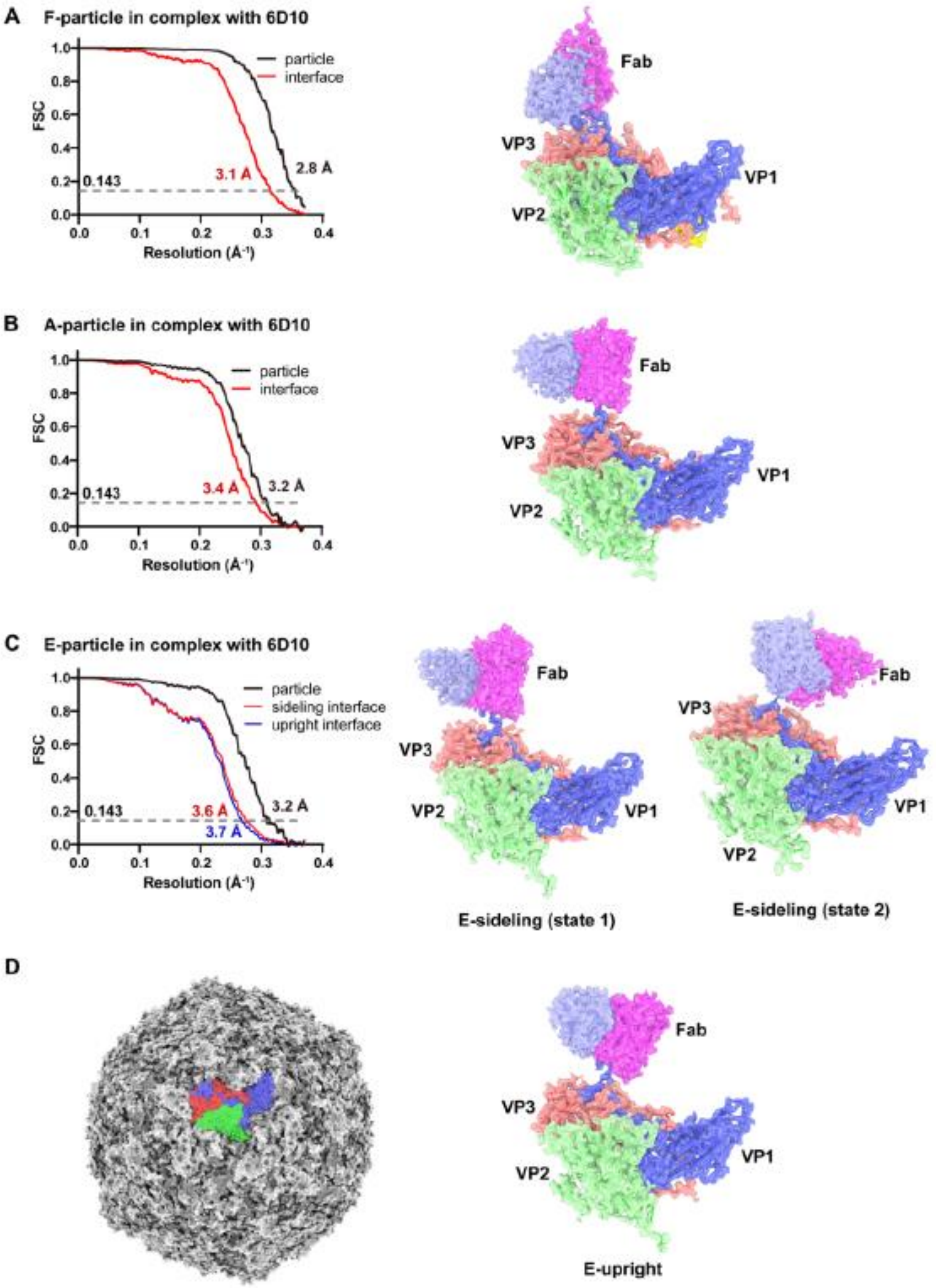

Figure S2. A-C. Cross-validation FSC curves for map-to-model fitting and the interfaces of E3 F-, A-, and E-particles with 6D10, respectively. VP1, VP2, and VP3 from one protomer are colored blue, green and red, respectively. Heavy chain and light chain of 6D10 are shown in purple and magenta, respectively. D. Surface representation of the E3 particle. A protomer located at the two-fold axes is highlight in the same color scheme.

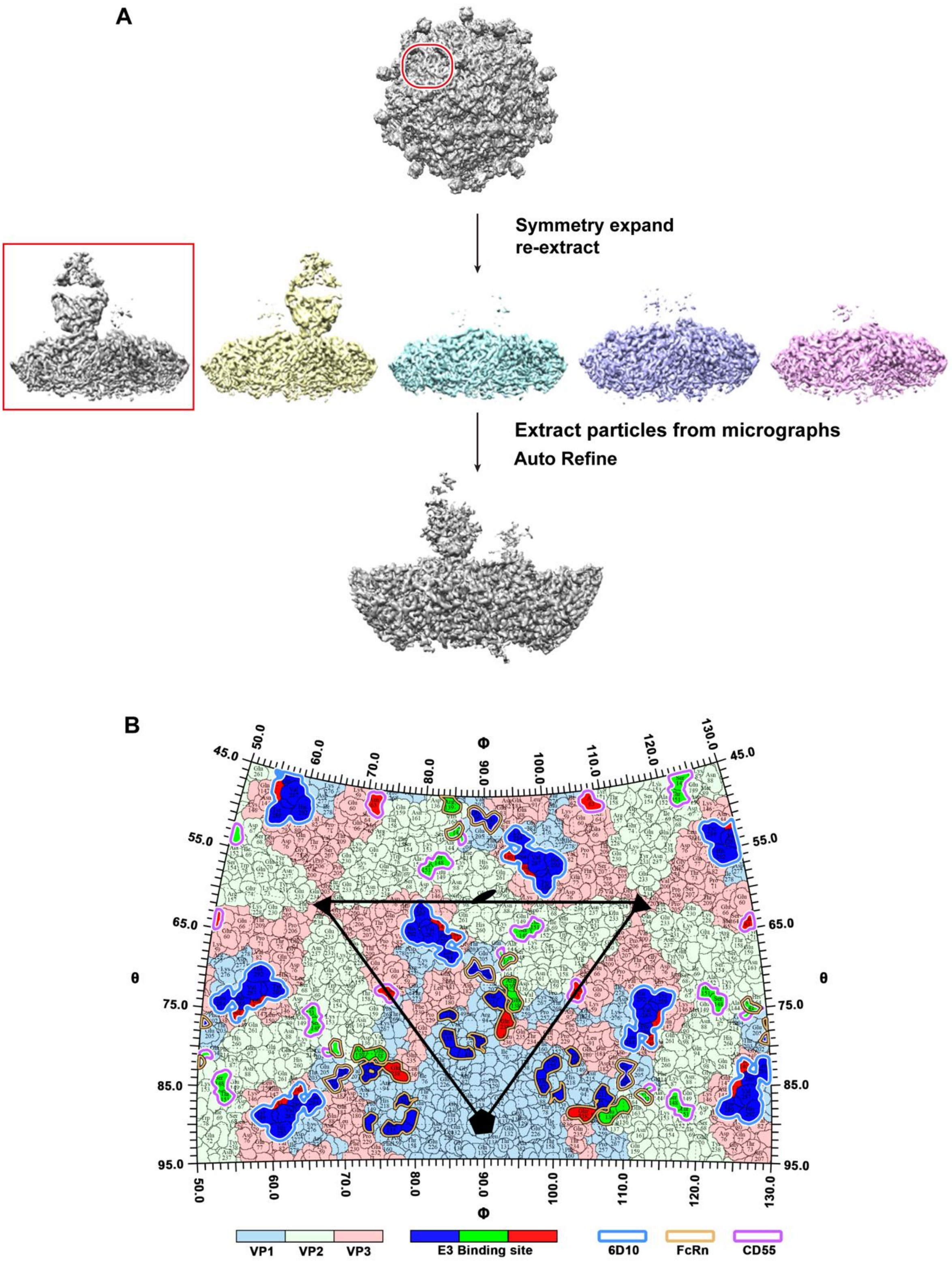

Figure S3. A. Data processing of E3-6D10 by BLOCK to improve the density at the interface. The red cycle indicates the unclear density of 6D10 before performing BLOCK. The best class obtained by BLOCK was highlighted by a red square, which was extracted again from the E3-6D10 particles, and further refined. B. The 6D10 footprints on the E3 surface. A two-dimensional projection of the E3 surface was produced using RIVEM. Residues of VP1, VP2, VP3 are colored in pale blue, pale green, pale pink, respectively. The residues involved in binding of E3 to 6D10 are colored blue,

green and red, respectively. The footprints of 6D10 and the receptors CD55 and FcRn are rimmed in light blue, purple and dark yellow, respectively. The triangles and five-pointed star indicate the two-, three-, and five-fold icosahedral symmetry axes.

Supplementary Table 1. Interactions of E3 with 6D10 (d < 4 Å)

**E3 F-particle**			**6D10**	
Location	Domain	Residues	Light chain	Heavy chain
VP1	C-terminus	L284	Y98	-
		P286	Y98, Y38, Y31	-
		V287	Y98	-
		Y289	-	N31
		H292	-	D57, N55
VP3	EF loop	S140	S32	-
		Q143	Y31	-
**E3 A-particle**			**6D10**	
Location	Domain	Residues	Light chain	Heavy chain
VP1	C-terminus	L284	Y98	-
		P286	Y98, Y31	-
		V287	-	T103
		H292	-	D57, N55
VP3	GH loop	Y184	Y31, S32, S33	-
**E3 E-upright**			**6D10**	
Location	Domain	Residues	Light chain	Heavy chain
VP1	C-terminus	L284	Y98	-
		P286	Y98, Y31	-
		V287	Y98	-
		H292	-	D57, N55
VP3	GH loop	Y184	Y31, S32	-
		D182	S32	-
**E3 E-sideling (state 1)**			**6D10**	
Location	Domain	Residues	Light chain	Heavy chain
VP1	C-terminus	P286	Y98, Y38, Y31	-
		V287	Y98	T103
		H292	-	D57, N55
**E3 E-sideling (state 2)**			**6D10**	
Location	Domain	Residues	Light chain	Heavy chain
VP1	C-terminus	K276	-	Q62
		L284	Y98	-
		P286	Y98, Y38, Y31	-
		V287	Y98	-
		D291	-	D57
		H292	-	D57
VP3	GH loop	Y184	Q27	-

Supplementary Table 2. Cryo-EM data collection and refinement statistics

	E3 F-particle with 6D10 (overall)	E3 A-particle with 6D10 (overall)	E3 E-particle with 6D10 (overall)	E3 F-particle with 6D10 (interface)	E3 A-particle with 6D10 (interface)	E3 E-particle with 6D10 (upright interface)	E3 E-particle with 6D10 (sideling interface)
**Data collection**							
Voltage (kV)				300			
Microscope				FEI Titan Krios			
Camera				K2 (Gatan)			
Electron exposure (e⁻/Å²)				30			
Number of frames collected per micrograph				25			
Automation software				SerialEM			
Defocus range (μm)				–1.2 to –2.5			
Pixel size (Å)				1.35			
**Overall map processing**							
Micrographs used	624	624	624	624	624	624	624
Symmetry imposed	I3	I3	I3	C1	C1	C1	C1
Initial particle images	18,206	18,206	18,206	325,260	183,060	115,500	115,500
Final particle images	5,421	3,051	1,925	65,284	55,687	24,188	22,712
Resolution at 0.143 FSC of masked reconstruction (Å)	2.8	3.2	3.2	3.1	3.4	3.7	3.6
Map sharpening B factor (Å²)	-92.85	-113.43	-90.31	-94.63	-99.28	-122.03	-96.55
**Local map refinement**							
Initial model used (PDB code)							
Refinement package	-	-	-	Phenix v1.19	Phenix v1.19	Phenix v1.19	Phenix v1.19
Model composition							
Non-hydrogen atoms	-	-	-	8,322	7,453	7,474	14,924
Protein residues	-	-	-	1,060	949	952	1,901
R.m.s. deviations							
Bond lengths (Å)	-	-	-	0.004	0.004	0.005	0.003
Bond angles (°)	-	-	-	0.58	0.62	0.68	0.633
B factors (Å²)							
Protein	-	-	-	45.99	60.5	52.22	41.12
Validation							
MolProbity score	-	-	-	1.84	1.67	1.72	1.74
Clashscore	-	-	-	7.77	6.57	5.81	6.84
Poor rotamers (%)	-	-	-	0	0	0	0
Ramachandran plot							
Favored (%)	-	-	-	93.79	95.63	94.06	94.73
Allowed (%)	-	-	-	6.21	4.37	5.94	5.22
Disallowed (%)	-	-	-	0	0	0	0.05
Cb outliers (%)	-	-	-	0	0	0	0
CaBLAM outliers (%)	-	-	-	3.91	3.12	3.11	3.56

Supplementary Table 3. Sequence of 6D10

Chain name	Sequence
Heavy chain of 6D10	EVQLQQSGPELARPWASVKISCQAFYTFSNYGMQWVKQSPGQG LEWIGPFYPGNADTSYNQKFKGKATLTADKSSSTAYMQFSSLTS EDSAVYYCARVVATTDFDYWGQGTTVTVSS
Light chain of 6D10	YIEASQSPSSLAVSVGEKVTMSCKSSQSLLYSSNQKNYLAWFQQ KPGQSPKLLIYWASTRESGVPDRFTGSGSGTDFTLTISSVKAEDL AVYYCQQYYSYPYTFGGGTKLKIKR
